# Physical activity patterns and socioeconomic position: the German National Health Interview and Examination Survey 1998 (GNHIES98)

**DOI:** 10.1186/1471-2458-12-1079

**Published:** 2012-12-15

**Authors:** Jonas D Finger, Thorkild Tylleskär, Thomas Lampert, Gert BM Mensink

**Affiliations:** 1 Department of Epidemiology and Health Reporting, Robert Koch Institute, General-Pape-Strasse. 62-66, Berlin, D-12101, Germany; 2 Centre for International Health, University of Bergen, Årstadveien 21, Bergen, N-5020, Norway

**Keywords:** Socioeconomic position, Education, Physical activity, Germany

## Abstract

**Background:**

We investigated the associations between education and leisure-time, occupational, sedentary and total physical-activity levels based on data from the German National Health Interview and Examination Survey 1998 (GNHIES98). The roles of income level, occupational status and other mediating variables for these associations were also examined.

**Methods:**

The total study sample of the GNHIES98 comprised 7,124 participants between the ages of 18 and 79. Complete information was available for 6,800 persons on leisure-time, sedentary and total physical-activity outcomes and for 3,809 persons in regular employment on occupational activity outcomes. The associations between educational level and physical activity (occupational, sedentary, leisure-time and total physical activity) were analysed separately for men and women using multivariate logistic regression analysis. Odds ratios (OR) of educational level on physical-activity outcomes were calculated and adjusted for age, region, occupation, income and other mediating variables.

**Results:**

After adjusting for age and region, a higher education level was associated with more leisure-time activity – with an OR of 1.6 (95% CI, 1.3-2.0) for men with secondary education and 2.1 (1.7-2.7) for men with tertiary education compared to men with primary education. The corresponding ORs for women were 1.3 (1.1-1.6) and 1.7 (1.2-2.4), respectively. Higher education was associated with a lower level of vigorous work activity: an OR of 6.9 (4.6-10.3) for men with secondary education and 18.6 (12.0-27.3) for men with primary education compared to men with tertiary education. The corresponding ORs for women were 2.8 (2.0-4.0) and 5.8 (4.0-8.5), respectively. Higher education was also associated with a lower level of total activity: an OR of 2.9 (2.2-3.8) for men with secondary education and 4.3 (3.3-5.6) for men with tertiary education compared to men with primary education. The corresponding ORs for women were 1.6 (1.2-2.0) and 1.6 (1.2-2.1), respectively.

**Conclusions:**

In Germany adults with a lower level of education are more physically active, both at work and overall, compared to adults with a higher education level, although they are less physically active in their leisure time. Higher work-related activity levels among adults with lower education may explain why they are less active in their leisure time.

## Background

Physical activity is an important factor in the prevention and treatment of non-communicable diseases [1,2]. Physical activity is defined as any bodily movement initiated by the work of skeletal muscles that increases energy expenditure beyond the basal level [3]. Many studies have focused on the health-enhancing effects of leisure-time physical activity, while the health implications of occupational physical activity and total physical activity (‘any bodily movement’) have rarely been investigated [4]. Epidemiological studies consistently show that the leisure-time physical-activity level is positively associated with the level of education, level of income, occupational status and summary indices of the socioeconomic position (SEP) [5-8]. Findings on the association between SEP and occupational and total physical-activity levels are inconsistent [5,9-12]. Some studies suggest that physical workers are less likely to participate in leisure-time physical activity [13-16] and show higher total activity levels [17,18] than sedentary workers. It might be interesting to know whether differences in the occupational activity level can explain why low-SEP groups show lower levels of leisure-time physical activity. This knowledge might be important in order to understand the social gradient of leisure-time physical activity, to design effective health-promotion interventions and to reduce health inequalities. The SEP aspect most frequently used in research on SEP and physical activity is the education level, since it has reduced temporal variation [7], and its response rate is generally higher compared to income and occupation. As far as we know, there is no study which uses all the main indicators of SEP (education, income and occupation) and investigates their mutual, interdependent effects on physical-activity indicators.

The aim of this study was to examine the associations between the level of education and leisure-time, occupational, sedentary and total activity levels among adults in Germany. Furthermore, it aimed to investigate the role of variables which mediate the association between education and physical activity, such as occupational status, income level, physical activity, ‘self-perceived health’, body mass index, smoking status and alcohol consumption. The comprehensive data on physical activity from the German National Health Interview and Examination Survey 1998 (GNHIES98) enabled us to investigate these associations.

## Methods

### Study design and participants

The GNHIES98 was a cross-sectional survey with data collected between October 1997 and March 1999 at 130 sample points distributed across the Federal Republic of Germany. The overall response rate of the GNHIES98 was 61.4%. A total of 7,124 participants were selected from population registries using a two-stage cluster sampling approach based on the sizes of the respective federal state and municipality; the participants were then selected stratified by age. The method is described in detail elsewhere [19]. The study population comprised the German-speaking adult population aged between 18 and 79. The survey was approved by the Board of the Federal Commissioner for Data Protection Berlin, Germany [20]. Each participant gave informed written consent before enrolling for the survey. Respondents were informed about the study objectives, the examination and interview process, and the applicable data protection guidelines (anonymous data processing and record keeping). All participants completed a self-administered health questionnaire and underwent a physical examination. Body height and weight were measured in a standardized way. Complete information was available on 6,800 respondents of the total study sample and 3,809 individuals who were currently working. The item response rate for all the specified variables was 97%.

### Definitions of variables

#### Physical activity

Four physical-activity outcome indicators were constructed based on the following questions. ‘*Vigorous work activity’*: ‘Is your present occupation characterized by vigorous physical activity (…)? Yes/No’ (assessed among respondents who indicated that they are ‘currently working’).

*‘Sports activity’*: ‘How often do you engage in sports: regularly, more than 4 hours per week; regularly, 2–4 hours per week; regularly, 1–2 hours per week; less than 1 hour per week; no sports activity?’ A dichotomous variable of sports activity ≥ 2 hours per week was constructed for analysis, since studies have shown that this approximate dose of leisure-time physical activity is related to an all-cause mortality-risk reduction of about 30% [4].

*‘High total energy expenditure’*: ‘How much time per day (24 hours) do you spend on average doing the following: (…) a) Sleeping, relaxing; b) Sitting down (…); c) Light activities (…); d) Moderately vigorous activities (…); e) Vigorous activities (…)?’ The question distinguishes between weekdays and weekend days. A 24-hour total-energy expenditure index was generated by assigning metabolic equivalent (MET) values [21,22] to the respective activity categories (sleeping = 0.9, sitting = 1.3, light activity = 2.5, moderately vigorous activity = 4.5, vigorous activity = 6 MET) and cumulating activities over 24 hours. One MET corresponds to the resting metabolic rate (RMR) and accounts for approximately 1 kcal/kg bodyweight per hour [22]. This short instrument only produces rough estimates of total energy expenditure and was used to rank individuals, instead of using the continuous MET scores for analysis. Total energy expenditure was further recoded into a binary variable for dividing the population into 60% versus 40% using the upper limit of the 3^rd^ quintile to define ‘energy expenditure’ as high (cut-off of 53 MET*hours per 24 hours for men and 48 for women). Schmidt et al. have compared a slightly revised version of the GNHIES98 total activity questionnaire with a comprehensive reference questionnaire and concluded that 82.5% of the study sample showed good or acceptable agreement between the questionnaires [23].

*‘Sitting time weekdays’* was generated using the item on time spent sitting on weekdays. Respondents were grouped into categories of sitting time by calculating quintiles of the ‘sitting time weekdays’ variable. Again, the cut-off used was the upper limit of the 3^rd^ quintile to define ‘sitting time weekdays’ as high (8 hours sitting time for both sexes). Rütten et al. have compared a similar ‘sitting time weekdays’ question with the question on sitting time asked in the International Physical Activity Questionnaire (IPAQ); they found a correlation coefficient of 0.6 [24].

#### Socioeconomic position (SEP)

*‘Education’* was assessed using two questions on the highest school-leaving certificate and the highest vocational-training certificate achieved by the respondent. A categorical education variable (primary, secondary, tertiary education) was generated by applying the ‘Comparative Analysis of Social Mobility in Industrial Nations’ (CASMIN) approach adapted to the German education system [25].

*‘Income’* was assessed using two questions about the households’ approximate monthly net income and the number of persons living permanently in the household. A household net equivalent income variable was generated by assigning need-specific weights (as recommended by OECD [26]) to the household members (head of household = 1, persons ≥ 15 years = 0.5, persons < 15 years = 0.3), calculating the household size, and dividing the monthly net income by the household size. A categorical income-level variable was created by grouping the household net equivalent income variable in tertiles.

*‘Occupation’* was assessed using one question on the current or last position of employment. A categorical occupational-status variable was generated according to a revised version of the ‘Occupational Prestige in Comparative Perspective’ approach for Germany, using the ‘scale of autonomy to act’ to categorize respondents into three categories of occupational status (low, middle, high) [27].

#### Covariates

*Body mass index* (BMI) was calculated on the basis of physical examination data on body weight and height [BMI = body weight (kg) / height (m)^2^]. A categorical BMI variable was calculated according to the guidelines of the World Health Organization (BMI <25, 25 - <30, ≥30).

*‘Self-perceived health’* was assessed with the question: ‘In general, would you say your health is: excellent; very good; good; fair; poor?’

*‘Smoking status’* was assessed in three categories: ‘current smoker’, ‘past smoker’ and ‘never smoked’.

*‘Alcohol consumption’* was assessed based on beverage-specific questions on the frequency and amount of drinks consumed. An alcohol index was constructed in terms of the grams of alcohol consumed per day. Respondents were grouped into categories of alcohol consumption by calculating quintiles of the alcohol index.

### Statistical analysis

The statistical analyses were performed using the software package STATA SE 11.0. In all statistical analyses, the cluster structure of the multi-stage sample was accounted for by using survey-design procedures. These adjustments lead to wider confidence intervals compared to those calculated for a simple random sampling setting. Unadjusted binary analyses were performed using logistic regression analyses. Confounding and interaction of covariates on the association between education level and physical-activity outcome variables was examined by performing stepwise-forward logistic regression analyses (*Model 1*: outcome and exposure variable; *Model 2*: Model 1 + covariate; *Model 3*: Model 2 + interaction term of exposure*covariate), storing the estimations of each model at each stage, and testing for model fit using a likelihood-ratio test (lrtest) – by comparing the post-estimations of the respective models. Confounding of a covariate was given if the lrtest was significant comparing the post-estimations of Model 1 and Model 2; interaction was given if additionally the lrtest was significant comparing Model 2 and Model 3. Associations between education level and physical-activity outcomes were analysed using stepwise-forward multiple logistic regression models, conducting a separate analysis for each outcome variable (occupational, sedentary, sports and total activity) the results were stratified by sex. The resulting odds ratios were used as indicators of the correlative strength of the association between education and physical activity. The age- and region-adjusted associations between education and physical-activity variables in the basic models were subsequently adjusted for occupation and income, as well as other significant confounders for the respective associations. When adjusting for covariates, we used the age strata 18–39, 40–59 and 60–79; the BMI categories < 25, 25–30 and > 30 kg/m^2^; the ‘self-perceived health’ strata ‘excellent’, ‘very good’, ‘good’, ‘fair’ and ‘poor’; the ‘work activity’ strata ‘vigorous work activity’ and ‘no vigorous work activity’; the ‘sports activity’ strata ‘no sports activity’, < 1, 1–2, 2–4 and > 4 hours per week; quintiles of the ‘sitting time weekdays’ and ‘alcohol consumption’ index; and the ‘smoking status’ categories ‘current smoker’, ‘past smoker’ and ‘never smoked’. Missing values of the income and occupation variables were included in the statistical analyses by generating a separate category for missing values (the numbers are shown in Table 1). Finally, subgroup analyses were performed, stratifying by identified effect-modifying variables.

**Table 1:** Selected variables of the participants aged 18–79 in the workforce subsample and the total sample in relation to key outcome variables

	**Workforce sample **^**a**^		**Vigorous work activity**	**Total sample**		**Sports ≥ 2 hours/week**	**Sitting time weekdays**	**Energy expenditure **^**b**^
	**n**	**%**	**%**	**n**	**%**	**%**	**mean hours**	**MET/24 hours**
**Total sample**	3809	100	40	6800	100	19	6.9	49.2
**Age group (years)**								
17-39	1898	50	42	2713	42	24	6.7	50.8
40-59	1788	47	38	2563	39	17	6.9	49.7
60-79	123	3	33	1524	24	11	6.8	45.4
**Sex**								
men	2109	55	43	3301	49	22	7.2	51.2
women	1700	45	36	3499	51	15	6.7	47.3
**Region in Germany**								
former East	1221	32	47	2312	34	13	6.8	50.1
former West	2588	68	37	4488	66	21	7.0	48.7
**Educational level**								
primary	1291	34	54	2929	43	14	6.5	49.4
secondary	1851	49	40	2921	43	21	6.9	50.0
tertiary	667	18	12	950	14	25	8.3	45.8
**Occupational status**								
low	1362	36	61	2675	39	13	6.1	51.0
middle	1387	36	29	2225	33	18	7.2	48.2
high	875	23	24	1260	19	23	7.9	47.5
missing	185	5	41	640	9	34	7.3	48.2
**Income level**								
low	864	23	53	1819	27	15	6.4	50.5
middle	1060	28	40	1856	27	19	6.8	49.7
high	1296	34	27	1796	26	24	7.8	47.6
missing	589	15	51	1329	20	16	6.6	48.8
**Body mass index**								
<25	1646	43	37	2705	40	24	6.9	49.0
25 - <30	1497	39	40	2648	39	18	6.9	49.7
≥30	654	17	46	1403	21	9	6.9	48.8
missing	12	<1		44	<1			
**Self-perceived health**								
excell./very good	936	25	33	1347	20	32	7.1	49.9
good	2440	64	41	4258	63	16	6.8	49.5
fair/poor	431	11	52	1192	17	11	7.1	47.0
missing	2	<1		3	<1			

^a^ Workforce sample: respondents indicating that they are ‘currently working’. ^b^ Energy expenditure assessed on the basis of self-reported activities within 24h, expressed in metabolic equivalents (MET) kcal/kg, 1 MET = a person’s caloric consumption at complete rest.

## Results

### Participants

The responder/non-responder analysis revealed that the responders were more likely to report a high level of education and better self-perceived health than the non-responders (see Additional file 1). The description of participants in the total study sample and the sub-study sample of the working population are presented in Table 1 in relation to selected variables as well as selected physical-activity outcome variables.

Table 2 shows the crude associations (odds ratios) between the outcome variables according to the exposure variable (‘education’) and other covariates used in the multivariate models. Total energy expenditure was higher in the workforce sample with a mean of 51.3 METs/24 hours, compared to the non-workforce sample with a mean of 46.5 (see Additional file 1: Table S7). Moreover, men and women in jobs characterized by vigorous work activity spent more time per 24 hours on weekdays engaging in moderate- and vigorous-intensity activities and less time in sedentary and light activities than other men and women (see Additional File 1: Table S8).

**Table 2:** Crude odds ratios (OR) of physical-activity indicators according to selected key variables, adults aged 18-79

	**No. in sample**^**a**^	**Vigorous work activity**	**No. in sample**^**b**^	**Sports ≥ 2 hours/week**	**Sitting time ≥ 8 hours/weekday**	**High energy expenditure**^**c**^
		**OR 95% CI**		**OR 95% CI**	**OR 95% CI**	**OR 95% CI**
**Total**	3809		6800			
**Education**						
primary	1291	8.6 (6.6-11.2)	2929	1.0	1.0	1.9 (1.6-2.3)
secondary	1851	4.8 (3.7- 6.3)	2921	1.6 (1.4-1.8)	1.3 (1.2-1.5)	2.0 (1.7-2.4)
tertiary	667	1.0	950	2.0 (1.6-2.4)	2.8 (2.3-3.3)	1.0
***Covariates***						
**Occupation**						
low	1362	5.0 (4.1-6.1)	2675	1.0	1.0	1.7 (1.4-1.9)
middle	1387	1.3 (1.1-1.6)	2225	1.5 (1.3-1.7)	2.0 (1.8-2.4)	1.0 (0.9-2.0)
high	875	1.0	1260	2.0 (1.7-2.3)	2.6 (2.2-3.1)	1.0
**Income**						
low	864	3.1 (2.5-3.7)	1819	1.0	1.0	1.7 (1.4-1.9)
middle	1060	1.8 (1.5-2.2)	1856	1.3 (1.1-1.6)	1.3 (1.1-1.5)	1.5 (1.3-1.7)
high	1296	1.0	1796	1.8 (1.6-2.1)	2.1 (1.8-2.5)	1.0
**Vigorous work activity**						
yes	1522	-	1557	1.0	1.0	6.5 (5.6-7.4)
no	2287	-	2319	1.6 (1.3-1.8)	4.5 (3.8-5.4)	1.0
**Sports activity** ≥ **2 hours/week**						
yes	778	1.0	1258	-	1.1 (0.9-1.3)	1.5 (1.3-1.7)
no	3031	1.6 (1.3-1.8)	5542	-	1.0	1.0
**Sitting time weekdays**						
≥ 8 hours	1564	1.0	1735	1.0 (0.9-1.2)	-	1.0
5 - < 8 hours	911	2.2 (1.8-2.7)	1929	1.0 (0.9-1.2)	-	6.4 (5.3-7.8)
< 5 hours	1334	6.8 (5.7-8.2)	2136	1.0	-	37 (31–46)
**Body mass index**						
<25	1646	1.0	2705	3.2 (2.6-3.9)	1.1 (1.0-1.3)	1.0
25 - <30	1497	1.1 (1.0-1.3)	2648	2.6 (1.8-2.8)	1.1 (0.9-1.2)	1.1 (1.0-1.3)
≥30	654	1.4 (1.2-1.7)	1403	1.0	1.0	1.0 (0.8-1.1)
**Self-perceived health**						
excell./very good	936	1.0	1347	4.0 (3.2-5.0)	1.1 (0.9-1.3)	1.6 (1.3-1.9)
good	2440	1.4 (1.2-1.7)	4258	1.6 (1.3-2.0)	0.9 (0.8-1.1)	1.5 (1.3-1.7)
fair/poor	431	2.3 (1.8-2.9)	1192	1.0	1.0	1.0

^a^ Number of observations in the workforce study sample (respondents indicating that they are ‘currently working’). ^b^ Number of observations in the total study sample. ^c^ Energy expenditure assessed on the basis of self-reported activities within 24h, expressed in metabolic equivalents (MET) , high energy expenditure = 4^th^ + 5^th^ quintiles of MET index.

### Multivariate analyses

#### Vigorous work activity

‘Sitting time weekdays’, ‘self-perceived health’ and ‘alcohol consumption’ were significant mediators (95% level of confidence) for the association between ‘education’ and ‘vigorous work activity’ (Table 3). After adjustment, a significant negative association remained between ‘education’ and ‘vigorous work activity’, and between ‘occupation’ and ‘vigorous work activity’ among both men and women.

**Table 3:** Stepwise adjusted odds ratios (OR) of vigorous work activity and sitting time weekdays among working men and women aged 18–79, by level of education

	**Vigorous work activity**		**Sitting time ≥ 8 hours/weekday**	
	**Model 1**^**a**^	**Final Model **^**b**^	**Model 1**^**a**^	**Final Model **^**c**^
	**OR 95%CI**	**OR 95%CI**	**OR 95%CI**	**OR 95%CI**
**Men (n=2109)**				
**Education**				
primary	18.6 (12.0-27.3)	5.5 (3.5-8.6)	1.0	1.0
secondary	6.9 (4.6-10.3)	3.4 (2.2-5.3)	2.1 (1.7-2.7)	1.5 (1.2-1.9)
tertiary	1.0	1.0	6.2 (4.7-8.3)	3.2 (2.4-4.3)
**Occupation**				
low		2.1 (1.5-3.1)		1.0
middle		1.0 (0.7-1.3)		2.3 (1.8-3.1)
high		1.0		2.4 (1.9-3.1)
**Income**				
low		1.3 (0.9-1.8)		1.0
middle		1.1 (0.8-1.5)		1.7 (1.3-2.4)
high		1.0		2.0 (1.5-2.7)
**Women (n=1700)**				
**Education**				
primary	5.8 (4.0-8.5)	2.8 (1.8-4.4)	1.0	1.0
secondary	2.8 (2.0-4.0)	1.9 (1.3-2.8)	1.6 (1.2-2.2)	1.1 (0.8-1.5)
tertiary	1.0	1.0	2.7 (1.9-3.8)	1.5 (1.0-2.3)
**Occupation**				
low		1.6 (1.1-2.5)		1.0
middle		1.0 (0.7-1.4)		2.9 (2.2-3.8)
high		1.0		1.8 (1.2-2.6)
**Income**				
low		1.0 (0.7-1.5)		1.0
middle		1.0 (0.7-1.3)		1.3 (0.9-1.7)
high		1.0		2.1 (1.6-2.8)

^a^ Model adjusted for age groups and regional strata East/West Germany. ^b^ Adjusted as Model 1 and also for sports activity, sitting time weekdays, self-perceived health and smoking status. ^c^ Adjusted as Model 1.

#### Sitting time ≥ 8 hours per weekday

No significant mediators were identified for the association between ‘education’ and ‘sitting time weekdays’ (Table 3). After inclusion of ‘occupation’ and ‘income’, significant positive associations were observed between all the SEP indicators and ‘sitting time weekdays’ ≥ 8 hours.

#### Sports activity ≥ 2 hours per week

‘Work activity’, BMI, ‘self-perceived health’ and ‘smoking status’ were significant mediators for the association between ‘education’ and ‘sports activity’ ≥ 2 hours per week (Table 4). The inclusion of work activity explained 38% of the association between ‘education’ and ‘sports activity’ among men and 75% among women, comparing people with primary and tertiary education. The corresponding percentages were 25% among men and 50% among women, comparing persons with primary and secondary education (see Additional file 1). After adjustment, a significant positive association between ‘income’ and ‘sports activity’ ≥ 2 hours per week remained among men and between ‘occupation’ and ‘sports activity’ ≥ 2 hours per week among women.

**Table 4:** Stepwise adjusted odds ratios (OR) of sports activity and total energy expenditure, men and women aged 18–79, by level of education

	**Sports ≥ 2 hours per week**		**High energy expenditure**	
	**Model 1**^**a**^	**Final Model **^**b**^	**Model 1**^**a**^	**Final Model **^**c**^
	**OR 95%CI**	**OR 95%CI**	**OR 95%CI**	**OR 95%CI**
**Men (n=3301)**				
**Education**				
primary	1.0	1.0	4.3 (3.3-5.6)	1.5 (0.9-2.3)
secondary	1.6 (1.3-2.0)	1.1 (0.9-1.5)	2.9 (2.2-3.8)	1.3 (0.9-2.0)
tertiary	2.1 (1.7-2.7)	1.2 (0.8-1.8)	1.0	1.0
**Occupation**				
low		1.0		1.5 (1.0-2.1)
middle		1.1 (0.8-1.4)		1.5 (1.1-2.2)
high		0.8 (0.6-1.2)		1.0
**Income**				
low		1.0		1.2 (0.9-1.7)
middle		1.6 (1.1-2.2)		1.2 (0.9-1.7)
high		1.9 (1.4-2.6)		1.0
**Women (n=3499)**				
**Education**				
primary	1.0	1.0	1.6 (1.2-2.1)	1.2 (0.7-2.0)
secondary	1.3 (1.1-1.6)	1.0 (0.7-1.4)	1.6 (1.2-2.0)	1.2 (0.8-1.8)
tertiary	1.7 (1.2-2.4)	0.9 (0.5-1.5)	1.0	1.0
**Occupation**				
low		1.0		0.6 (0.4-1.0)
middle		1.2 (0.9-1.8)		0.8 (0.5-1.1)
high		1.8 (1.1-2.8)		1.0
**Income**				
low		1.0		1.5 (0.9-2.3)
middle		0.8 (0.6-1.2)		1.2 (0.8-1.8)
high		0.9 (0.6-1.3)		1.0

^a^ Model adjusted for age groups and regional strata East/West Germany. ^b^ Adjusted as Model 1 and also for work activity, body mass index, perceived health, alcohol consumption ^c^ Adjusted as Model 1 and also for work activity, sitting time, body mass index, perceived health.

#### High total energy expenditure

‘Work activity’, ‘sitting time weekdays’, BMI and ‘self-perceived health’ were found to be significant mediators for the association between ‘education’ and ‘high total energy expenditure’ (Table 4). After adjustment, a negative association between ‘occupation’ and ‘high total energy expenditure’ remained among men.

### Subgroup analyses

Age was found to be a significant effect modifier for the associations between ‘education’ and ‘sports activity’ ≥ 2 hours per week, ‘high total energy expenditure’ and ‘sitting time weekdays’ ≥ 8 hours. In the 18–59 age group (Table 5), the association between ‘education’ and ‘sports activity’ ≥ 2 hours per week was weaker than in the 60–79 age group. The association between ‘education’ and ‘sitting time weekdays’ ≥ 8 hours per weekday was positive and significant in the 18–59 age group; no association was observed in the 60–79 age group, however. The association between ‘education’ and ‘high total energy expenditure’ was negative in the 18–59 age group and positive in the 60–79 age group.

**Table 5:** Crude analysis of the association between education and physical-activity indicators, stratified by age group

	**No. in sample**	**Sports activity ≥ 2 hours/week**	**Sitting time ≥ 8 hours/weekday**	**High total energy expenditure **^**a**^
		**OR 95% CI**	**OR 95% CI**	**OR 95% CI**
**Total**	6800			
**18-59 years of age**	5276			
Primary education	1824	1.0	1.0	1.0
Secondary education	2654	1.3 (1.1-1.5)	1.6 (1.4-1.9)	0.7 (0.6-0.9)
Tertiary education	798	1.7 (1.3-2.1)	3.7 (3.0-4.5)	0.3 (0.3-0.4)
**60-79 years of age**	1524			
Primary education	1105	1.0	1.0	1.0
Secondary education	267	2.2 (1.5-3.3)	0.8 (0.6-1.0)	1.6 (1.1-2.2)
Tertiary education	152	2.6 (1.7-4.0)	1.0 (0.7-1.4)	1.2 (0.8-1.7)

^a^ Energy expenditure assessed on the basis of self-reported activities within 24h, expressed in metabolic equivalents (MET) kcal/kg, 1 MET = a person’s caloric consumption at complete rest, high energy expenditure = 4^th^ + 5^th^ quintiles of MET index.

The ‘sports activity’ variable was found to be an effect modifier for the associations between ‘education’ and ‘high total energy expenditure’, and between ‘education’ and ‘vigorous work activity’. The stratified analysis revealed that both of the specified associations were stronger among participants reporting no sports activity than among respondents reporting sports activity ≥ 2 hours per week (data not shown).

The variable on whether respondents were ‘currently working’ was found to be an effect modifier for the associations between ‘education’ and ‘sitting time weekdays’, as well as between ‘education’ and ‘total energy expenditure’. Both associations were stronger among people who were currently working than among people who were not working, especially in the case of men (data not shown).

## Discussion

In this nationwide cross-sectional study of a randomly selected sample of adults in Germany, it is observed that adults with a lower level of education are more physically active when they are working, less active in leisure time, and expend more energy in total than adults with a higher education level. These observations are in line with the findings of other studies [11,13,15-18]. Education shows the strongest independent associations with the physical-activity variables compared to occupation and income; this also confirms the findings of previous studies [5].

Studies suggest that individuals who engage in more vigorous activity during working hours may, as a response, have lower levels of physical activity during leisure time and vice versa [14-16]. We found that the association between education and leisure-time activity is mediated by vigorous work activity, which supports the hypothesis that low-SEP groups are less physically active in their leisure time because they are more physically active when working. It should be born in mind however, that some people are more active over all domains than others. 8% of adults with primary education, 7 with secondary education and 2.5 with tertiary education reported simultaneously that they fulfil vigorous working tasks and engage in sports activity at least 2 hours per week. Another mediator for the association between education and leisure-time activity is BMI. A high body mass index can act as a barrier to engaging in leisure-time activity, and the higher prevalence of overweight among people with a lower level of education might partly explain the education disparities in leisure-time activity.

Occupational physical activity dominates the 24-hour total energy-expenditure index because occupational activity usually corresponds to an 8-hour working day, and leisure-time physical activity is usually performed over shorter periods [28]. Thus, high-SEP individuals who are mainly sedentary at work may be unable to fully offset the higher energy expended at work by individuals with a lower SEP. A representative study from the United States based on accelerometer data has observed that men and women with active jobs have more wear-time counts in total than men and women with sedentary jobs [17]. The same database indicated that men with a lower education level had more wear-time counts on average than men with a higher level of education [29], which is in line with our results.

The described patterns change in higher age groups, where SEP-related differences in work-related activity tend to disappear. In this higher age group, the association between education and sports activity was stronger, the association between education and sitting time weekdays disappeared, and the negative association between education and high total energy expenditure turned into a positive association. It may be that individuals who used to do physically-active jobs during their working years do not increase their participation in sports activity when they retire, with the result that their level of total energy expenditure decreases. This may have something to do with the fact that they have not been socialized to participate in leisure-time physical activity to maintain a good level of physical fitness and may lack networks, opportunities and the physical health to start doing so at an older age when they stop working. By contrast, adults with higher education who may have compensated inactivity at work with leisure-time physical activity during their working years may use their increased free time in retirement to increase level of leisure-time physical activity.

Social cognitive theory distinguishes different types of behaviour according to the degree of volitional control an individual has in order to change behaviour. Non-volitional behaviour is defined as behaviour over which the individual has limited freedom of control, whereas volitional behaviour is a form of behaviour over which the individual does have the freedom of control [30]. It may be reasonable to categorize occupational activity as non-volitional behaviour, since the individual has only limited freedom to change occupational activities that are determined by contract. Thus, work-related activity might be seen as a structural factor which influences leisure-time activity behaviour. Occupational activities are directly related to a person’s educational background, occupational position and level of income. Hence, the associations between SEP variables and occupational activities are particularly strong. The stronger the freedom to control behaviour, the weaker might be its socioeconomic determination. Perhaps this explains why the socioeconomic prediction of leisure-time activity was found to be lower than for occupational activity.

An important question may arise when considering our results: if physical activity is good for a person’s health, and adults with a higher level of education are less physically active in total, why are they healthier? In addition to a healthier diet and lower smoking prevalence, one hypothesis may be that occupational physical activity does not have the same health benefits as leisure-time physical activity. Most studies showing a reduction of mortality risk with increasing physical activity rely on leisure-time activity data [4]. Findings on occupational physical activity and health are inconsistent, but in studies that assess both leisure-time and occupational activity, the dose–response relationship tends to be lower for occupational activity than for leisure-time activity [15,31-36]. Two studies which investigated the relationship between physically demanding work and physical fitness showed that, although muscular strength was greater among physical workers than among sedentary workers, aerobic fitness was higher among sedentary workers in one study but showed no difference in the other [37,38]. Lakka et al. has also shown that occupational energy expenditure is not positively related to cardiorespiratory fitness among Finnish men [39]. Although physically demanding work tasks vary widely depending upon the occupation [21], it appears that work activity often correlates with muscle-strengthening activity (e.g. lifting and carrying heavy things), and leisure-time activity with aerobic physical activity (e.g. running, cycling, swimming). Muscle-strengthening activity and aerobic physical activity do not generate the same physiological adaptations and health benefits [40,41], which may partially explain why low-SEP groups show lower cardiovascular health than high-SEP groups, even though they expend more energy in total.

Although the magnitudes of the SEP gradients on domain-specific activities may have changed since 1998 [42], we assume that the results of the study are still relevant, since the SEP gradient on physical-activity patterns has remained fairly consistent over the years [42,43].

### Limitations

No causal inferences can be drawn when interpreting these results, since the study relies on cross-sectional data, and physical activity was assessed on the basis of self-reports. The GNHIES98 is a general health survey. It was not possible to assess physical activity in a comprehensive way. Total physical activity was only assessed using five intensity categories of physical activity with a distinction between weekdays and weekend days. The total activity questionnaire therefore produces rather rough estimates of total energy expenditure on a MET basis. As a result, it was decided to use the information obtained to rank individuals – rather than the continuous MET values – as the outcome for analysis. Studies show that physical activity questionnaires overestimate the duration and intensity of physical activity compared to objective measurements such as the accelerometer or the doubly labelled water method [44,45], and there is evidence to suggest that the internal validity of questionnaires is lower than that of accelerometers when defining health risks based on biomarkers [46]. Social desirability bias – as well as cognitive problems relating to recalling the durations of activity and categorizing the intensity of activities – compromise the internal validity of self-reported physical activity information [47,48]. Reporting bias is particularly problematic if it differs systematically according to specific characteristics of the respondents, causing differential misclassification bias [49]; we cannot totally exclude this possibility in this study. The average MET score of total energy expenditure revealed here also seemed to be rather high. This may be partly attributable to the oversampling of formerly East German people in GNHIES98. Individuals who lived in the former East Germany were oversampled by 30% in order to increase the statistical power for East–west comparisons. In 1998, the economic structures of the former East and West Germany were different, since the ‘Fall of the Berlin Wall’ had taken place only ten years earlier in 1989. The economy in the former East Germany had large agricultural and industrial sectors involving high levels of physically demanding work, while the former West German economy had a large service industry sector with sedentary office jobs. In Germany as a whole, the service-industry sector has grown significantly since 1998, although the structural changes have been stronger in the former East than in western Germany. Furthermore, the prevalence of sports activities has also increased in Germany since 1998. A recent trend study demonstrated that the prevalence of performing ‘any’ sports activity had increased in Germany from 56.0% in 1998 to 61.4% in 2003 and 64.6% in 2009 [42]. However, studies also demonstrate that the SEP gradient on physical-activity patterns has not essentially changed in Germany in the last fifteen years [42,43].

The physical activity questionnaire used in this study differs from those used in other countries, which makes it difficult to compare results [24].

## Conclusions

It is important to assess physical activity using setting-specific approaches which distinguish between leisure-time and work-related physical activity when investigating social inequalities in physical-activity behaviour. The social gradient is fundamentally different in the work and leisure domains of physical activity; low-SEP groups are more active at work, high-SEP groups more so in their leisure time. The low leisure-time physical-activity level among lower-SEP groups seems not only to be the result of individual preferences, but also to depend on structural determinants. Physically demanding work is a structural factor which seems to be partly responsible for the lower participation of low-SEP groups in leisure-time physical activity. Health-promotion interventions focusing on increasing leisure-time physical activity should consider the structural factor of physically demanding work, which can act as a barrier to engaging in leisure-time physical-activity programmes. Furthermore, retired people who used to do physically demanding work during their working years should be an important focus group for health-enhancing physical-activity programmes. They need assistance in developing strategies to compensate the loss of daily physical activity due to the end of physical demanding work tasks when they stop working.

## Competing interests

The GNHIES98 was financed by the German Federal Ministry of Health. The study was conducted by the Robert Koch Institute, which is a federal institute within the portfolio of the Federal Ministry of Health. The authors declare that they have no competing interests.

## Authors’ contributions

JF analysed and structured the data, and wrote both the first draft and the final version of the manuscript. GM was involved in the design of GNHIES98, developed the physical activity questions and constructed the total energy expenditure index. TL contributed to constructing the socioeconomic position variables. TT contributed to structuring the statistical analyses. All authors contributed to writing and revising the manuscript, and read and approved the final manuscript.

## Pre-publication history

The pre-publication history for this paper can be accessed here:

http://www.biomedcentral.com/1471-2458/12/1079/prepub

## Supplementary Material

Additional file 1 **The ‘Additional File’ (in pdf format) contains the results of the responder/non-responder analysis (Table 6), the differences between the workforce and non-workforce samples as regards physical-activity outcome variables (Table 7), the mean hours stated for weekday activities in the total activity questionnaire – by physical work status and education (Table 8) – as well as detailed information on intermediate steps undertaken during logistic regression analyses (Tables 9–12).** Adobe Acrobat Reader is required to access this file. Click here for file
