# Supplementary material for: Physical activity patterns and socioeconomic position: the German National Health Interview and Examination Survey 1998 (GNHIES98)
Source: BMC Public Health. 2012 Dec 15;12:1079. doi: 10.1186/1471-2458-12-1079 (PMC3561273; doi:10.1186/1471-2458-12-1079)
Supplement: Additional file 1 — The ‘Additional File’ (in pdf format) contains the results of the responder/non-responder analysis (Table 6), the differences between the workforce and non-workforce samples as regards physical-activity outcome variables (Table 7), the mean hours stated for weekday activities in the total activity questionnaire – by physical work status and education (Table 8) – as well as detailed information on intermediate steps undertaken during logistic regression analyses (Tables 9–12). Adobe Acrobat Reader is required to access this file. [file 1471-2458-12-1079-S1.doc]

**Additional File**

| **Table 6. Differences between responders and non-responders, adults aged 18-79, by selected variables** | | | | | |
| --- | --- | --- | --- | --- | --- |
|  | **Responders**  **(n=6800)** |  | **Non-responders**  **(n=1832)** |  | **Level of significance** ^a^ |
| **Education (%)** |  |  |  |  |  |
| Left school with no qualification certificate | 1.9 (1.6-2.2) |  | 0.8 (0.3-1.1) |  | P = 0.001 |
| Secondary modern school/ elementary school-leaving certificate | 40.4 (39.3-41.6) |  | 50.1 (47.8-52.4) |  | P < 0.001 |
| Secondary school certificate | 19.2 (18.2-20.1) |  | 20.2 (18.4-22.0) |  | P = 0.32 |
| Polytechnic upper school certificate 10^th^ grade (before 1965: 8^th^ grade) | 14.0 (13.2-14.9) |  | 8.7 (7.4-10.0) |  | P < 0.001 |
| Advanced level (college of further education certificate) | 6.3 (5.8-6.9) |  | 3.6 (2.7-4.5) |  | P < 0.001 |
| Advanced level, general advanced level or in specialized subjects (grammar school or upper school) | 16.0 (15.1-16.9) |  | 13.5 (11.9-15.0) |  | P < 0.01 |
|  |  |  |  |  |  |
| **State of health** |  |  |  |  |  |
| Self-perceived health “good” or better (%) | 82.5 (81.5-83.3) |  | 62.3 (60.1-64.5) |  | P < 0.001 |
| Mean days sick in the last 12 months | 18.5 (15.7-21.4) |  | 12.5 (11.6-13.4) |  | P < 0.001 |
|  |  |  |  |  |  |
| **Smoking status (%)** |  |  |  |  |  |
| Daily smoker | 26.6 (25.5-27.6) |  | 28.0 (25.9-30.0) |  | P = 0.22 |
| Occasional smoker | 6.4 (5.8-7.0) |  | 6.9 (5.7-8.0) |  | P = 0.45 |
| Non-smoker | 67.1 (65.9-68.2) |  | 65.1 (63.0-67.3) |  | P = 0.12 |
|  |  |  |  |  |  |
| **Body mass index (means) ^b^** | 26.7 (26.5-26.8) |  | 25.5 (25.3-25.7) |  | P < 0.001 |
|  |  |  |  |  |  |
| **Living together with partner (%)** | 75.0 (74.0-76.1) |  | 68.9 (66.7-71.0) |  | P < 0.001 |
| ^a^ Test of proportion was performed for binary outcomes, t-test for continuous outcomes. **^b^** Body mass index = weight (kg) / height (m)². Note: Height and body weight were assessed based on self-reports among the non-responders and objectively measured for the responders. Self-reported estimates on height and weight tend to underestimate the body mass index. | | | | | |

| **Table 7. Differences between workforce and non-workforce respondents as regards physical-activity outcome variables, men and women aged 18-79** | | | | | |
| --- | --- | --- | --- | --- | --- |
|  | No. in sample | **Vigorous work activity** | **Sports** ≥ **2 hours/week** | **Sitting time weekdays in hours/day** | **Total energy expenditure** ^a^ **in METs/24 hours** |
|  |  | % 95%CI | % 95%CI | mean 95%CI | mean 95%CI |
| **Workforce** |  |  |  |  |  |
| men | 2109 | 43.2 (40.4-46.0) | 23.4 (21.3-25.5) | 7.2 (7.0-7.4) | 53.3 (52.3-54.1) |
| women | 1700 | 35.9 (33.3-38.6) | 16.8 (14.9-18.6) | 6.9 (6.7-7.1) | 48.9 (48.3-49.5) |
| total | 3809 | 40.0 (37.7-42.2) | 20.4 (19.0-21.9) | 7.1 (6.9-7.3) | 51.3 (50.6-52.0) |
| **Others** |  |  |  |  |  |
| men | 1192 | - | 20.6 (17.8-23.3) | 7.1 (6.9-7.3) | 47.6 (46.9-48.3) |
| women | 1799 | - | 13.1 (11.3-14.9) | 6.5 (6.3-6.7) | 45.8 (45.3-46.2) |
| total | 2991 | - | 16.0 (14.4-17.7) | 6.7 (6.6-6.9) | 46.5 (46.1-46.9) |
| **Whole sample** |  |  |  |  |  |
| men | 3301 | - | 22.4 (20.5-24.2) | 7.2 (7.0-7.3) | 51.2 (50.5-51.9) |
| women | 3499 | - | 14.9 (13.5-16.2) | 6.7 (6.6-6.9) | 47.3 (46.9-47.7) |
| total | 6800 | - | 18.6 (17.2-19.8) | 6.9 (6.8-7.1) | 49.2 (48.7-49.6) |
| Abbreviations: CI, confidence interval. ^a^ Energy expenditure assessed on the basis of self-reported activities within 24h, expressed in metabolic equivalents (MET) kcal/kg, 1 MET = a person’s caloric consumption at complete rest. | | | | | |

| **Table 8. Weekday activities in the 24-hour physical activity questionnaire, by physical work status and education** | | | | | | |
| --- | --- | --- | --- | --- | --- | --- |
|  | No. in sample | **Sleeping hours/day** | **Sitting hours/day** | **Light activity hours/day** | **Moderately vigorous activity hours/day** | **Vigorous activity hours/day** |
|  |  | mean 95%CI | mean 95%CI | mean 95%CI | mean 95%CI | mean 95%CI |
| **Physical work status** |  |  |  |  |  |  |
| vigorously working men | 911 | 7.2 (7.1-7.2) | 5.3 (5.1-5.5) | 3.3 (3.1-3.4) | 4.2 (4.0-4.4) | 4.1 (3.8-4.3) |
| other men | 2390 | 7.7 (7.7-7.8) | 7.9 (7.7-8.0) | 4.6 (4.4-4.7) | 2.5 (2.3-2.6) | 1.4 (1.3-1.5) |
| vigorously working women | 611 | 7.4 (7.4-7.5) | 5.4 (5.2-5.7) | 5.3 (5.1-5.5) | 3.5 (3.3-3.7) | 2.3 (2.0-2.5) |
| other women | 2888 | 8.0 (7.9-8.0) | 7.0 (6.8-7.1) | 5.8 (5.7-5.9) | 2.5 (2.4-2.6) | 0.7 (0.7-0.8) |
|  |  |  |  |  |  |  |
| **Education** |  |  |  |  |  |  |
| men with primary education | 1389 | 7.7 (7.7-7.8) | 6.5 (6.3-6.7) | 4.3 (4.2-4.5) | 3.0 (2.9-3.2) | 2.4 (2.2-2.6) |
| men with tertiary education | 603 | 7.6 (7.5-7.6) | 8.8 (8.4-9.1) | 4.5 (4.3-4.8) | 2.0 (1.8-2.2) | 1.1 (1.0-1.3) |
| women with primary education | 1540 | 8.0 (7.9-8.1) | 6.5 (6.3-6.7) | 5.8 (5.6-5.9) | 2.7 (2.6-2.8) | 1.0 (0.9-1.1) |
| women with tertiary education | 347 | 7.6 (7.5-7.7) | 7.8 (7.4-8.1) | 5.5 (5.2-5.8) | 2.4 (2.1-2.6) | 0.8 (0.6-0.9) |

Abbreviations: CI, confidence interval.

| **Table 9. Stepwise-adjusted odds ratios of vigorous work activity, men and women aged 18-79, by education** | | | | | |
| --- | --- | --- | --- | --- | --- |
|  | **Vigorous work activity** | | | | |
|  | Basic model ^a^; age, region, education | Model 1; basic model + occupation, income | Model 2 ^b^; model 1 + sports activity | Model 3 ^c^; model 2 + sitting time weekdays | Model 4 ^d^; model 3 + self-perceived health + smoking status |
|  | OR 95%CI | OR 95%CI | OR 95%CI | OR 95%CI | OR 95%CI |
| **Men**  **(n=2109)** |  |  |  |  |  |
| **Education** |  |  |  |  |  |
| primary | 18.6 (12.4-27.8) | 8.5 (5.6-12.9) | 7.7 (5.1-11.8) | 5.7 (3.6-9.0) | 5.5 (3.5-8.6) |
| secondary | 6.9 (4.6-10.3) | 4.4 (2.9-6.6) | 4.2 (2.8-6.3) | 3.4 (2.2-5.2) | 3.4 (2.2-5.3) |
| tertiary | 1.0 | 1.0 | 1.0 | 1.0 | 1.0 |
|  |  |  |  |  |  |
| **Occupation** |  |  |  |  |  |
| missing |  | 1.5 (1.0-2.2) | 1.7 (1.1-2.6) | 1.8 (1.2-2.8) | 1.8 (1.2-2.9) |
| low |  | 2.7 (2.0-3.7) | 2.7 (1.9-3.6) | 2.1 (1.5-3.1) | 2.1 (1.5-3.1) |
| middle |  | 1.0 (0.7-1.3) | 1.0 (0.7-1.3) | 1.0 (0.7-1.3) | 1.0 (0.7-1.3) |
| high |  | 1.0 | 1.0 | 1.0 | 1.0 |
|  |  |  |  |  |  |
| **Income** |  |  |  |  |  |
| missing |  | 1.7 (1.2-2.6) | 1.6 (1.1-2.4) | 1.4 (0.9-2.1) | 1.4 (0.9-2.0) |
| low |  | 1.7 (1.2-2.3) | 1.6 (1.2-3.6) | 1.3 (0.9-1.8) | 1.3 (0.9-1.8) |
| middle |  | 1.1 (0.9-1.5) | 1.1 (0.9-1.5) | 1.1 (0.8-1.4) | 1.1 (0.8-1.5) |
| high |  | 1.0 | 1.0 | 1.0 | 1.0 |
|  |  |  |  |  |  |
| **Women (n=1700)** |  |  |  |  |  |
| **Education** |  |  |  |  |  |
| primary | 5.8 (4.0-8.5) | 3.7 (2.4-5.7) | 3.5 (2.3-5.4) | 3.0 (2.0-4.7) | 2.8 (1.8-4.4) |
| secondary | 2.8 (2.0-4.0) | 2.3 (1.6-3.4) | 2.3 (1.5-3.3) | 2.0 (1.4-2.9) | 1.9 (1.3-2.8) |
| tertiary | 1.0 | 1.0 | 1.0 | 1.0 | 1.0 |
|  |  |  |  |  |  |
| **Occupation** |  |  |  |  |  |
| missing |  | 0.8 (0.4-1.5) | 0.8 (0.5-1.5) | 1.0 (0.5-1.8) | 1.0 (0.6-1.8) |
| low |  | 1.8 (1.2-2.7) | 1.7 (1.1-2.6) | 1.6 (1.1.2.4) | 1.6 (1.1-2.5) |
| middle |  | 0.8 (0.6-1.1) | 0.8 (0.6-1.1) | 1.0 (0.7-1.4) | 1.0 (0.7-1.4) |
| high |  | 1.0 | 1.0 | 1.0 | 1.0 |
|  |  |  |  |  |  |
| **Income** |  |  |  |  |  |
| missing |  | 1.7 (1.3-2.4) | 1.7 (1.2-2.3) | 1.4 (1.0-1.9) | 1.4 (1.0-2.0) |
| low |  | 1.4 (1.0-1.9) | 1.3 (0.9-1.8) | 1.0 (0.7-1.5) | 1.0 (0.7-1.5) |
| middle |  | 1.2 (0.9-1.6) | 1.2 (0.9-1.6) | 1.0 (0.7-1.3) | 1.0 (0.7-1.3) |
| high |  | 1.0 | 1.0 | 1.0 | 1.0 |
| Abbreviations: OR, odds ratio; CI, confidence interval. ^a^ Age-group strata used: 18-39, 40-59 and 60-79; regional strata: former East/West Germany. ^b^ Sports activity strata used: no sports activity, < 1, 1-2, 2-4 and >4 hours per week. ^c^ Sitting time weekdays strata used (quintiles): very low, low, middle, high and very high. ^d^ Self-perceived health strata used: excellent, very good, good, fair and poor; smoking status strata used: never, current, past. | | | | | |

| **Table 10. Stepwise-adjusted odds ratios of sitting time ≥ 8 hours/weekday, men and women aged 18-79, by education** | | | | | |
| --- | --- | --- | --- | --- | --- |
|  | **Sitting time ≥ 8 hours/weekday** | | | | |
|  | Basic model ^a^; age, region, education | Model 1; basic model + occupation, income | Model 2 ^b^; model 1 + sports activity | Model 3 ^c^; model 2 + work activity | Model 4 ^d^; model 3 + body mass index + self-perceived health |
|  | OR 95%CI | OR 95%CI | OR 95%CI | OR 95%CI | OR 95%CI |
| **Men**  **(n=3301)** |  |  |  |  |  |
| **Education** |  |  |  |  |  |
| primary | 1.0 | 1.0 | 1.0 | 1.0 | 1.0 |
| secondary | 1.6 (1.3-2.0) | 1.3 (1.1-1.6) | 1.3 (1.1-1.6) | 1.3 (1.0-1.7) | 1.3 (1.0-1.7) |
| tertiary | 3.5 (2.8-4.4) | 2.4 (1.9-3.0) | 2.5 (1.9-3.1) | 2.1 (1.6-3.0) | 2.3 (1.6-3.1) |
|  |  |  |  |  |  |
| **Occupation** |  |  |  |  |  |
| low |  | 1.0 | 1.0 | 1.0 | 1.0 |
| middle |  | 1.5 (1.2-1.9) | 1.5 (1.2-1.9) | 1.8 (1.3-2.4) | 1.8 (1.4-2.4) |
| high |  | 1.6 (1.3-2.0) | 1.6 (1.3-2.1) | 1.9 (1.4-2.5) | 1.9 (1.4-2.5) |
| missing |  | 2.2 (1.6-3.0) | 2.3 (1.7-3.2) | 2.6 (1.6-4.3) | 2.7 (1.6-4.5) |
|  |  |  |  |  |  |
| **Income** |  |  |  |  |  |
| low |  | 1.0 | 1.0 | 1.0 | 1.0 |
| middle |  | 1.2 (1.0-1.5) | 1.2 (1.0-1.5) | 1.6 (1.1-2.1) | 1.6 (1.2-2.2) |
| high |  | 1.5 (1.2-1.8) | 1.5 (1.2-1.9) | 1.8 (1.3-2.4) | 1.8 (1.3-2.4) |
| missing |  | 1.0 (0.8-1.3) | 1.0 (0.8-1.3) | 1.1 (0.8-1.7) | 1.1 (0.8-1.7) |
|  |  |  |  |  |  |
| **Women (n=3499)** |  |  |  |  |  |
| **Education** |  |  |  |  |  |
| primary | 1.0 | 1.0 | 1.0 | 1.0 | 1.0 |
| secondary | 1.3 (1.1-1.5) | 1.0 (0.8-1.2) | 1.0 (0.9-1.2) | 1.0 (0.8-1.4) | 1.0 (0.8-1.4) |
| tertiary | 2.1 (1.6-2.8) | 1.4 (1.1-1.9) | 1.5 (1.1-2.1) | 1.3 (0.8-1.9) | 1.3 (0.8-1.9) |
|  |  |  |  |  |  |
| **Occupation** |  |  |  |  |  |
| low |  | 1.0 | 1.0 | 1.0 | 1.0 |
| middle |  | 1.8 (1.5-2.2) | 1.9 (1.5-2.3) | 2.6 (1.9-3.5) | 2.6 (1.9-3.5) |
| high |  | 1.4 (1.1-1.9) | 1.5 (1.1-1.9) | 1.6 (1.1-2.4) | 1.6 (1.1-2.4) |
| missing |  | 2.3 (1.7-3.0) | 2.4 (1.8-3.2) | 2.6 (1.7-4.2) | 2.6 (1.6-4.1) |
|  |  |  |  |  |  |
| **Income** |  |  |  |  |  |
| low |  | 1.0 | 1.0 | 1.0 | 1.0 |
| middle |  | 1.1 (0.9-1.3) | 1.1 (0.9-1.4) | 1.2 (0.9-1.7) | 1.2 (0.9-1.7) |
| high |  | 1.7 (1.4-2.2) | 1.8 (1.4-2.2) | 2.1 (1.6-2.8) | 2.1 (1.6-2.8) |
| missing |  | 1.1 (0.8-1.3) | 1.0 (0.8-1.3) | 1.1 (0.8-1.6) | 1.1 (0.8-1.6) |
| Abbreviations: OR, odds ratio; CI, confidence interval. ^a^ Age-group strata used: 18-39, 40-59 and 60-79; regional strata: former East/West Germany. ^b^ Sports activity strata used: no sports activity, < 1, 1-2, 2-4 and >4 hours per week. ^c^ Work activity strata used: vigorous activity and no vigorous activity. ^d^ Body mass index strata used: < 25, 25-30 and > 30 kg/m²; self-perceived health strata used: excellent, very good, good, fair and poor. | | | | | |

| **Table 11. Stepwise-adjusted odds ratios of sports activity ≥ 2 hours/week, men and women aged 18-79, by education** | | | | | |
| --- | --- | --- | --- | --- | --- |
|  | **Sports ≥ 2 hours/week** | | | | |
|  | Basic model ^a^; age, region, education | Model 1; basic model + occupation, income | Model 2 ^b^; model 1 + work activity | Model 3 ^c^; model 2 + body mass index | Model 4 ^d^; model 3 + self-perceived health + alcohol consumption |
|  | OR 95%CI | OR 95%CI | OR 95%CI | OR 95%CI | OR 95%CI |
| **Men**  **(n=3301)** |  |  |  |  |  |
| **Education** |  |  |  |  |  |
| primary | 1.0 | 1.0 | 1.0 | 1.0 | 1.0 |
| secondary | 1.6 (1.3-2.0) | 1.3 (1.1-1.7) | 1.2 (1.0-1.6) | 1.2 (0.9-1.6) | 1.1 (0.9-1.5) |
| tertiary | 2.1 (1.7-2.7) | 1.7 (1.3-2.3) | 1.4 (1.0-2.1) | 1.4 (0.9-2.0) | 1.2 (0.8-1.8) |
|  |  |  |  |  |  |
| **Occupation** |  |  |  |  |  |
| low |  | 1.0 | 1.0 | 1.0 | 1.0 |
| middle |  | 1.2 (0.9-1.5) | 1.1 (0.8-1.5) | 1.1 (0.8-1.4) | 1.1 (0.8-1.4) |
| high |  | 1.2 (0.9-1.5) | 0.9 (0.6-1.2) | 0.9 (0.6-1.2) | 0.8 (0.6-1.2) |
| missing |  | 3.1 (2.3-4.2) | 2.8 (1.6-4.8) | 2.8 (1.6-4.8) | 2.5 (1.5-4.2) |
|  |  |  |  |  |  |
| **Income** |  |  |  |  |  |
| low |  | 1.0 | 1.0 | 1.0 | 1.0 |
| middle |  | 1.5 (1.2-1.9) | 1.5 (1.1-2.1) | 1.5 (1.1-2.2) | 1.6 (1.1-2.2) |
| high |  | 1.7 (1.3-2.2) | 1.9 (1.4-2.6) | 1.9 (1.4-2.6) | 1.9 (1.4-2.6) |
| missing |  | 1.2 (0.9-1.5) | 1.2 (0.8-1.7) | 1.1 (0.8-1.7) | 1.2 (0.8-1.7) |
|  |  |  |  |  |  |
| **Women (n=3499)** |  |  |  |  |  |
| **Education** |  |  |  |  |  |
| primary | 1.0 | 1.0 | 1.0 | 1.0 | 1.0 |
| secondary | 1.3 (1.1-1.6) | 1.2 (0.9-1.5) | 1.1 (0.8-1.6) | 1.0 (0.8-1.4) | 1.0 (0.7-1.4) |
| tertiary | 1.7 (1.2-2.4) | 1.3 (0.9-2.0) | 1.1 (0.7-1.9) | 1.0 (0.6-1.6) | 0.9 (0.5-1.5) |
|  |  |  |  |  |  |
| **Occupation** |  |  |  |  |  |
| low |  | 1.0 | 1.0 | 1.0 | 1.0 |
| middle |  | 1.2 (1.0-1.5) | 1.3 (0.9-1.8) | 1.2 (0.9-1.7) | 1.2 (0.9-1.8) |
| high |  | 1.5 (1.1-2.1) | 1.9 (1.2-2.9) | 1.8 (1.1-2.8) | 1.8 (1.1-2.8) |
| missing |  | 2.0 (1.5-2.7) | 2.1 (1.2-3.6) | 1.9 (1.1-3.3) | 1.9 (1.1-3.4) |
|  |  |  |  |  |  |
| **Income** |  |  |  |  |  |
| low |  | 1.0 | 1.0 | 1.0 | 1.0 |
| middle |  | 1.0 (0.8-1.4) | 0.9 (0.6-1.3) | 0.9 (0.6-1.3) | 0.8 (0.6-1.2) |
| high |  | 1.2 (1.0-1.6) | 1.0 (0.7-1.4) | 0.9 (0.7-1.3) | 0.9 (0.6-1.3) |
| missing |  | 1.0 (0.7-1.3) | 1.1 (0.7-1.7) | 1.1 (0.7-1.7) | 1.0 (0.6-1.5) |
| Abbreviations: OR, odds ratio; CI, confidence interval. ^a^ Age-group strata used: 18-39, 40-59 and 60-79; regional strata: former East/West Germany. ^b^ Work-activity strata used: vigorous activity and no vigorous activity. ^c^ Body mass index strata used: < 25, 25-30 and > 30 kg/m². ^d^ Self-perceived health strata used: excellent, very good, good, fair and poor; alcohol index strata used (quintiles): very low, low, middle, high and very high. | | | | | |

| **Table 12.** **Stepwise-adjusted odds ratios of high total energy expenditure, men and women aged 18-79, by education** | | | | | |
| --- | --- | --- | --- | --- | --- |
|  | **High total energy expenditure (men ≥ 53 and women ≥ 48 MET/hours per 24 hours)** | | | | |
|  | Basic model ^a^; age, region, education | Model 1; basic model + occupation, income | Model 2 ^b^; model 1 + work activity | Model 3 ^c^; model 2 + sitting time weekdays | Model 4 ^d^; model 3 + body mass index + self-perceived health |
|  | OR 95%CI | OR 95%CI | OR 95%CI | OR 95%CI | OR 95%CI |
| **Men**  **(N=3301)** |  |  |  |  |  |
| **Education** |  |  |  |  |  |
| primary | 4.3 (3.3-5.6) | 2.8 (2.1-3.8) | 2.3 (1.6-3.3) | 1.5 (1.0-2.3) | 1.5 (0.9-2.0) |
| secondary | 2.9 (2.2-3.8) | 2.4 (1.8-3.2) | 1.8 (1.3-2.6) | 1.4 (0.9-2.1) | 1.3 (0.9-2.3) |
| tertiary | 1.0 | 1.0 | 1.0 | 1.0 | 1.0 |
|  |  |  |  |  |  |
| **Occupation** |  |  |  |  |  |
| missing |  | 0.7 (0.5-1.0) | 0.9 (0.5-1.5) | 1.0 (0.4-2.0) | 1.0 (0.5-2.1) |
| low |  | 1.7 (1.4-2.2) | 1.9 (1.4-2.5) | 1.4 (1.0-2.1) | 1.5 (1.0-2.1) |
| middle |  | 1.2 (0.9-1.5) | 1.4 (1.0-1.8) | 1.5 (1.0-2.1) | 1.5 (1.1-2.2) |
| high |  | 1.0 | 1.0 | 1.0 | 1.0 |
|  |  |  |  |  |  |
| **Income** |  |  |  |  |  |
| missing |  | 1.3 (1.0-1.7) | 1.3 (0.9-1.8) | 0.9 (0.6-1.2) | 0.9 (0.6-1.2) |
| low |  | 1.3 (1.1-1.6) | 1.7 (1.2-2.2) | 1.2 (0.8-1.7) | 1.2 (0.8-1.7) |
| middle |  | 1.4 (1.1-1.7) | 1.2 (1.0-1.6) | 1.2 (0.9-1.7) | 1.2 (0.9-1.7) |
| high |  | 1.0 | 1.0 | 1.0 | 1.0 |
|  |  |  |  |  |  |
| **Women (N=3499)** |  |  |  |  |  |
| **Education** |  |  |  |  |  |
| primary | 1.6 (1.2-2.1) | 1.4 (1.0-1.8) | 1.3 (0.9-1.9) | 1.1 (0.7-1.8) | 1.2 (0.7-2.0) |
| secondary | 1.6 (1.2-2.0) | 1.5 (1.1-2.0) | 1.3 (1.0-1.9) | 1.1 (0.7-1.8) | 1.2 (0.7-1.8) |
| tertiary | 1.0 | 1.0 | 1.0 | 1.0 | 1.0 |
|  |  |  |  |  |  |
| **Occupation** |  |  |  |  |  |
| missing |  | 0.7 (0.5-0.9) | 0.6 (0.3-1.0) | 0.7 (0.3-1.4) | 0.7 (0.3-1.3) |
| low |  | 1.0 (0.7-1.2) | 0.9 (0.6-1.3) | 0.6 (0.4-1.0) | 0.6 (0.4-1.0) |
| middle |  | 0.7 (0.5-0.9) | 0.6 (0.4-0.8) | 0.8 (0.5-1.1) | 0.8 (0.5-1.1) |
| high |  | 1.0 | 1.0 | 1.0 | 1.0 |
|  |  |  |  |  |  |
| **Income** |  |  |  |  |  |
| missing |  | 1.3 (1.0-1.7) | 1.2 (0.9-1.7) | 0.7 (0.5-1.2) | 0.7 (0.5-1.1) |
| low |  | 1.6 (1.3-2.1) | 2.0 (1.4-2.8) | 1.4 (0.9-2.3) | 1.5 (0.9-2.3) |
| middle |  | 1.4 (1.1-1.8) | 1.7 (1.3-2.3) | 1.2 (0.8-1.8) | 1.2 (0.8-1.8) |
| high |  | 1.0 | 1.0 | 1.0 | 1.0 |
| Abbreviations: OR, odds ratio; CI, confidence interval; MET, metabolic equivalent kcal/kg, 1 MET = a person’s caloric consumption at complete rest. ^a^ Age-group strata used: 18-39, 40-59 and 60-79; regional strata: former East/West Germany. ^b^ Work-activity strata used: vigorous activity and no vigorous activity. ^c^ Sitting time weekdays strata (quintiles) used: very low, low, middle, high and very high. ^d^ Body mass index strata used: < 25, 25-30 and > 30 kg/m²; self-perceived health strata used: excellent, very good, good, fair and poor. | | | | | |
